# Using Machine Learning to Predict Mortality for COVID-19 Patients on Day 0 in the ICU

**DOI:** 10.3389/fdgth.2021.681608

**Published:** 2022-01-13

**Authors:** Elham Jamshidi, Amirhossein Asgary, Nader Tavakoli, Alireza Zali, Soroush Setareh, Hadi Esmaily, Seyed Hamid Jamaldini, Amir Daaee, Amirhesam Babajani, Mohammad Ali Sendani Kashi, Masoud Jamshidi, Sahand Jamal Rahi, Nahal Mansouri

**Affiliations:** ^1^Functional Neurosurgery Research Center, Shohada Tajrish Comprehensive Neurosurgical Center of Excellence, Shahid Beheshti University of Medical Sciences, Tehran, Iran; ^2^Department of Biotechnology, College of Sciences, University of Tehran, Tehran, Iran; ^3^Trauma and Injury Research Center, Iran University of Medical Sciences, Tehran, Iran; ^4^Department of Clinical Pharmacy, School of Pharmacy, Shahid Beheshti University of Medical Sciences, Tehran, Iran; ^5^Department of Genetic, Faculty of Advanced Science and Technology, Tehran Medical Sciences, Islamic Azad University, Tehran, Iran; ^6^School of Mechanical Engineering, Sharif University of Technology, Tehran, Iran; ^7^Department of Pharmacology, School of Medicine, Shahid Beheshti University of Medical Sciences, Tehran, Iran; ^8^Master of Business Administration (MBA)-University of Tehran, Tehran, Iran; ^9^Department of Exercise Physiology, Tehran University, Tehran, Iran; ^10^Swiss Institute for Experimental Cancer Research (ISREC), School of Life Sciences, École Polytechnique Fédérale de Lausanne (EPFL), Lausanne, Switzerland; ^11^Division of Pulmonary Medicine, Department of Medicine, Lausanne University Hospital (CHUV), University of Lausanne (UNIL), Lausanne, Switzerland; ^12^Laboratory of the Physics of Biological Systems, Institute of Physics, École Polytechnique Fédérale de Lausanne (EPFL), Lausanne, Switzerland

**Keywords:** SARS-CoV-2, COVID-19, artificial intelligence, ICU—intensive care unit, machine learning (ML)

## Abstract

**Rationale:** Given the expanding number of COVID-19 cases and the potential for new waves of infection, there is an urgent need for early prediction of the severity of the disease in intensive care unit (ICU) patients to optimize treatment strategies.

**Objectives:** Early prediction of mortality using machine learning based on typical laboratory results and clinical data registered on the day of ICU admission.

**Methods:** We retrospectively studied 797 patients diagnosed with COVID-19 in Iran and the United Kingdom (U.K.). To find parameters with the highest predictive values, Kolmogorov-Smirnov and Pearson chi-squared tests were used. Several machine learning algorithms, including Random Forest (RF), logistic regression, gradient boosting classifier, support vector machine classifier, and artificial neural network algorithms were utilized to build classification models. The impact of each marker on the RF model predictions was studied by implementing the local interpretable model-agnostic explanation technique (LIME-SP).

**Results:** Among 66 documented parameters, 15 factors with the highest predictive values were identified as follows: gender, age, blood urea nitrogen (BUN), creatinine, international normalized ratio (INR), albumin, mean corpuscular volume (MCV), white blood cell count, segmented neutrophil count, lymphocyte count, red cell distribution width (RDW), and mean cell hemoglobin (MCH) along with a history of neurological, cardiovascular, and respiratory disorders. Our RF model can predict patient outcomes with a sensitivity of 70% and a specificity of 75%. The performance of the models was confirmed by blindly testing the models in an external dataset.

**Conclusions:** Using two independent patient datasets, we designed a machine-learning-based model that could predict the risk of mortality from severe COVID-19 with high accuracy. The most decisive variables in our model were increased levels of BUN, lowered albumin levels, increased creatinine, INR, and RDW, along with gender and age. Considering the importance of early triage decisions, this model can be a useful tool in COVID-19 ICU decision-making.

**Graphical Abstract:**
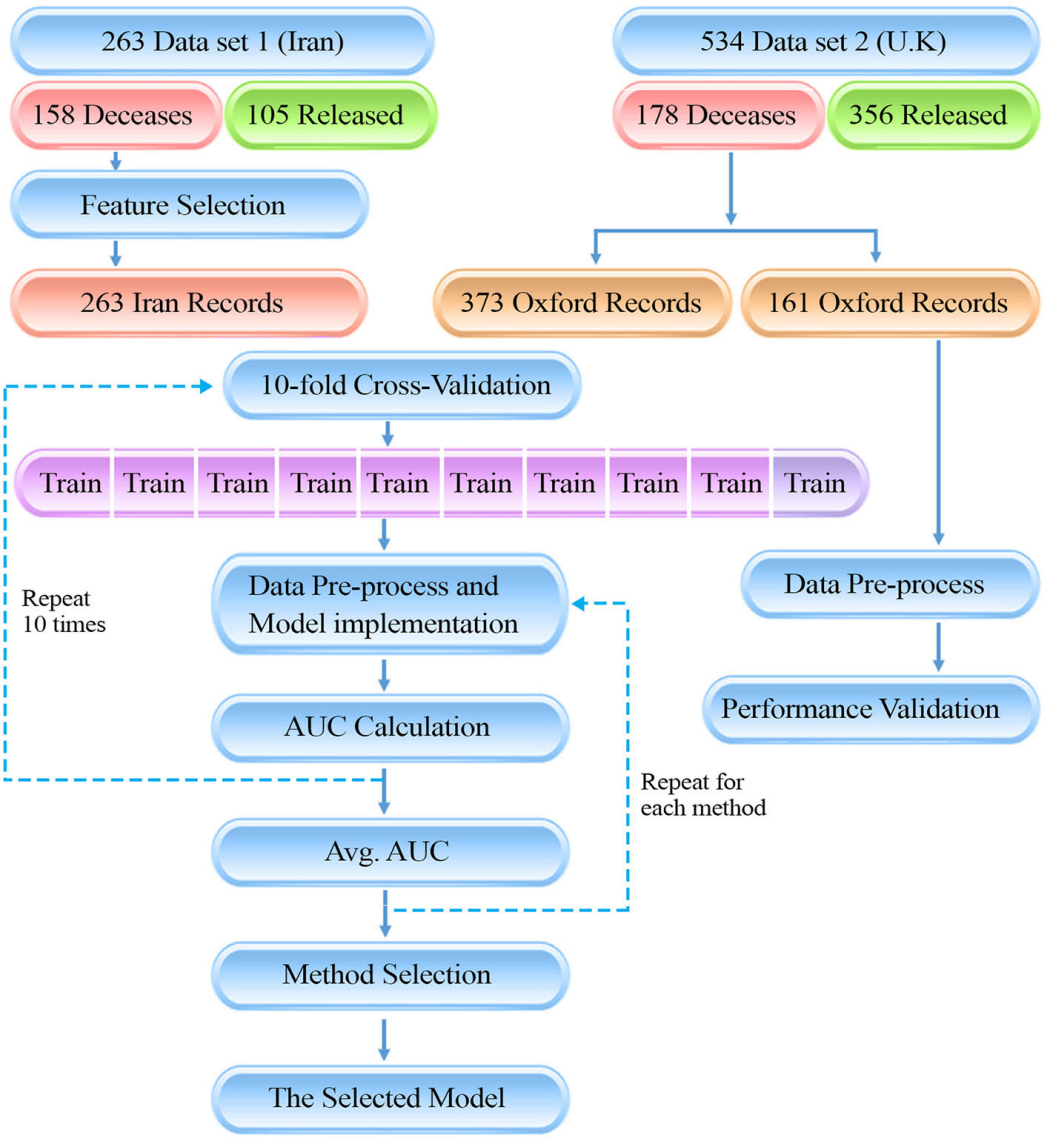
The presenting diagram, is showing the flow of our data gathering and method for the study. There are two data sources which ultimately have been used in a 10-fold cross-validation method to train the machine learning models. Finally, the model with the highest AUC was selected as final model.

## Introduction

As of September 6, 2021, COVID-19 has caused more than 219 million infections worldwide and resulted in more than 4.55 million deaths. Complications are more common among elderly patients and people with preexisting conditions, and the rate of intensive care unit (ICU) admission is substantially higher in these groups (1, 2).

ICU admissions rely on the critical care capacity of the health care system. Iran, which is the primary testbed for this study, was one of the first countries hit by COVID-19. The ICU admission rate involves about 32% of all hospitalizations, and the ICU mortality rate is about 39% (3). With the potential of new waves of COVID-19 infections driven by more transmissible variants, ICU hospitalization numbers are expected to rise, leading to shortages of ICU beds and critical management equipment. There is also the risk of a global shortage of effective medical supplies, making the judicious use of these medications a top priority for healthcare systems.

An individual-based prediction model is essential for tailoring treatment strategies and would aid in expanding our insights into the pathogenesis of COVID-19. A number of risk assessment scores are available to predict the severity of different diseases in ICU patients (4). Predictors of the need for intensive respiratory or vasopressor support in patients with COVID-19 and of mortality in COVID-19 patients with pneumonia have been identified (5, 6). To date, no general mortality prediction scores have been available for ICU admitted COVID-19 patients, irrespective of the patients' clinical presentation. Additionally, existing risk scales rely on parameters measured by health care providers such as blood pressure, respiratory rate, and oxygen saturation, which are subject to human error and operator bias especially under challenging and stressful conditions when numbers of COVID-19 patients surge (7). Thus, it remains vital to develop more unbiased risk-assessment tools that can predict the most likely outcomes for individual patients with COVID-19.

Recent advances in artificial intelligence (AI) technology for disease screening show promise as computer-aided diagnosis and prediction tools (8–11). In the era of COVID-19, AI has played an important role in early diagnosis of infection, contact tracing, and drug and vaccine development (12). Thus, AI represents a useful technology for the management of COVID-19 patients with the potential to help control the mortality rate of this disease. Nevertheless, an AI tool for making standardized and accurate predictions of outcomes in COVID-19 patients with severe disease is currently missing.

Beyond the general benefits of data-driven decision-making, the pandemic has also exposed the need for computational assistance to health care providers, who under the pressure of severely ill patients may make mistakes in judgment (7, 13, 14). Stressful conditions and burnout in health care providers can reduce their clinical performance, and a lack of accurate judgment can lead to increased mortality rates (15, 16). Artificial intelligence can help healthcare professionals determine who needs a critical level of care more precisely. Indeed, the effective use of AI could mitigate the severity of this outbreak.

Here, we propose a personalized machine-learning (ML) method for predicting mortality in COVID-19 patients based on routinely available laboratory and clinical data on the day of ICU admission.

## Methods

### Data Resources

This is an international study involving patients from Iran (dataset 1) and the United Kingdom (U.K., dataset 2). We retrospectively studied 797 adult patients with severe COVID-19 infection confirmed through reverse transcription-polymerase chain reaction (RT-PCR). Two hundred sixty-three patients were admitted to ICUs at different hospitals in Tehran, Iran between February 19 and May 1, 2020, and 534 patients were admitted to ICUs and Emergency Assessment Units based on the Oxfordshire Research Database. The study was performed after approval by the Iran University of Medical Sciences Ethics Committee (approval ID: IR.IUMS.REC.1399.595).

### Development of Mortality Prediction Model Using

The Mortality prediction model was aimed to predict whether patients were deceased or got released at the end of the admission period. Due to the generalizability and accessibility of the predictors recorded for patients in dataset 1 (Iran), and to reduce the model's feature space dimensionality, we merely used this dataset (consisting of 263 patients) for feature selection and model development. Only parameters with the highest predictive values were used in the modeling, leading to more robustness and generalizability of the model (17). Aside from that, further ML comparisons and validation was done with both the dataset 1 and 2.

### Statistical Analysis and Feature Selection

On the day of the ICU admission, 66 parameters were assessed for each patient including 11 demographic characteristics (e.g., age and gender), past medical history and comorbidities (including nine different preexisting conditions), and 55 laboratory biomarkers. These parameters are listed in Table 1. Sixty-nine percent of measurements were reported on the day of admission, 27% were reported 1 day after, and 4% were reported within 2 days of ICU admission because of sampling limitations and laboratory practice. We excluded patients whose laboratory data were obtained more than 2 days after the date of admission to the ICU.

**Table 1:** Machine learning methods with their parameters.

**Method**	**Parameter**	**Value**
Random forest	Number of trees	50
	Min. number of samples at a leaf node	0.1% of all samples
	Criterion	Gini
Logistic regression	C	1.0
Gradient boosting	Number of boosting stages to perform	10
	Fraction of samples used for fitting individual base learners	0.8
	Min. number of samples at a leaf node	10% of all samples
	Number of iterations with no change required for early stopping	3
	Max. number of features considered when looking for a split	3
Support vector machine	C	1.0
	Kernel type	RBF
	Kernel coefficient	1/number of features
Artificial neural network	Number of hidden layers	3
	Output space dimensionality for each hidden layer	32, 16, 8
	Activation function for each layer	Tanh, tanh, tanh, sigmoid

The aim was to predict a patient's survival. For the selection of parameters with the highest predictive value, under the null hypothesis of distributions being the same between the two groups, the two-sample Kolmogorov-Smirnov test (KS), shown in Supplementary Figure 1, was used for numerical parameters (age and laboratory biomarkers), and the Pearson chi-squared test (χ^2^), shown in Supplementary Figure 2, was used for categorical parameters (e.g., gender and comorbidities).

All selected predictors were available in the second dataset (Oxfordshire, U.K.). Henceforth, the datasets have been merged, solely possessing previously selected predictors in common.

To investigate multicollinearity, Variance Inflation Factor (VIF) was calculated for each predictor and reported in Supplementary Table 1. A cut-off of 10 has been used to omit predictors that are showing collinearity, which includes none of the included predictors.

### Data Preprocessing

Due to the difference in the measurement units and the necessity of units to be uniform, measurements of numerical parameters were unified between the two data sets by applying appropriate conversion factors, resulting in admissible input parameters for the model.

Data processing was carried out in four steps: First, because of incomplete laboratory data and in order to reduce difficulties associated with missing values, 771 patients out of the 797 total patients were selected as they had the data of at least 70 percent of all the biomarkers. Patients that did not have enough data present for biomarkers were removed. Second, samples were randomly separated into 10 independent sets with stratification over outcomes for 10-fold cross-validation to ensure the generalizability of the models (18). Of the 10 subsets, a single subset was retained as a validation set for model testing and the remaining nine subsets were used as training data. The cross-validation process was then iterated 10 times with each of the 10 subsets being used as the validation data exactly once. Third, numerical parameters were standardized by scaling the features to mean zero and unit variance. Last, missing biomarker values were imputed using the k-nearest neighbor (k-NN) algorithm, and a binary indicator of missingness for each biomarker was added to the dataset (17, 19). Standardization and imputation were performed separately on each cross-validation iteration by using training set samples.

### Machine Learning Model

**Figure 1:**
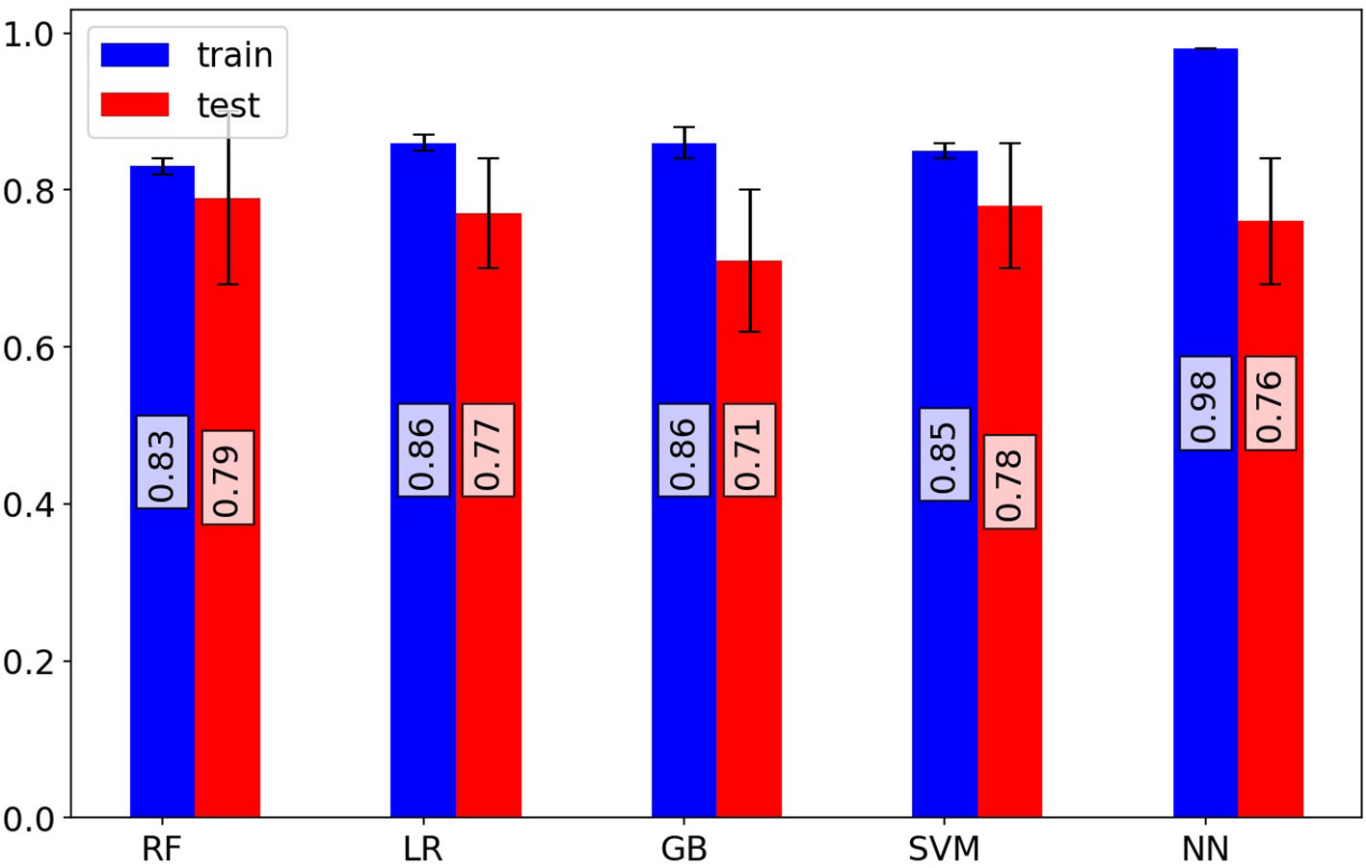
Investigation of model performance. Mean area under the receiver operating characteristic curve (ROC-AUC) of random forest, logistic regression, gradient boosting classifier, support vector machine classifier, and artificial neural network models for training and test sets of cross-validation iterations. The random forest model shows superior performance on validation sets. The random forest model predicts patient outcomes with a 70% sensitivity and 75% specificity.

**Figure 2:**
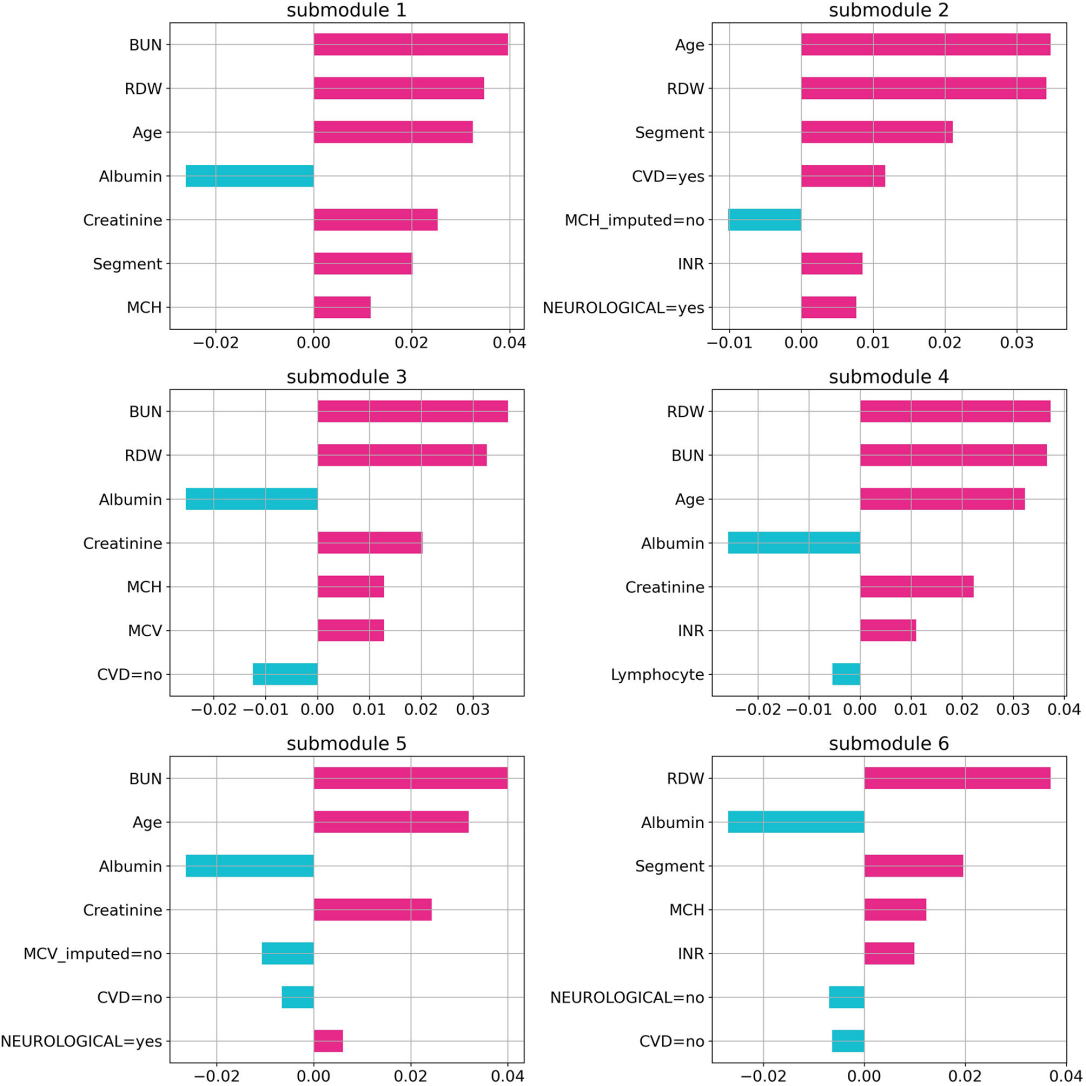
Feature importance in random forest model. The importance of the random forest features using local interpretable model-agnostic explanation submodular-pick with six submodules. Each submodule is related to a patient subpopulation (six subpopulation in this case) and represents decision criteria for them in the model. Negative values (blue) indicate favorable parameters suggesting a better prognosis, and positive values (red) indicate unfavorable parameters suggesting a worse prognosis.

Random forest (RF), logistic regression (LR), gradient boosting (GB), support vector machine (SVM), and artificial neural network (NN) methods were used to build classification models using the Python scikit-learn package. Methods along with their parameters are listed in Table 1. The performance of each method on training and validation sets in each cross-validation iteration was compared using a receiver operating characteristic curve (ROC), which is shown in Supplementary Figure 3. Area Under the Curve of ROC for each method is represented in Figure 1. Additional evaluation metrics for each model are also reported in Supplementary Table 2. To prevent overfitting in the training process, the LR model was trained with an L2 regularization factor equal to one, and the RF was forced to hold more than 10% of samples in each of its terminal leaves (20, 21). The statistically significant difference between models' AUC curves has been affirmed by DeLong's test and corresponding DeLong's *p*-values assure the RF model's superiority and are shown in Supplementary Figure 4. To find the most influential parameters in the LR model prediction, we used regression coefficients, which are shown in the Supplementary Figure 5. Using the local interpretable model-agnostic explanation submodular-pick (LIME-SP) method, we identified different patterns among the whole feature space in the RF model (22). The LIME-SP method can interpret the model's predictions in different parts of the feature space by modeling a subset of model predictions in the feature space around the sample with the help of linear models that are more interpretable. In our study, LIME-SP was performed on 100 random samples to find six submodules with the most disparity in their selected markers, as shown in Figure 2. To identify meaningful clinical differences between patients, seven parameters with the highest predictive values were derived from each submodule.

### Evaluation Criteria of the Model

To specify the evaluation dataset required for the validation of the model's performance, 30% of the records available in dataset 2 (the U.K, 161 patients; equal to 20% of the records) were randomly selected and assigned to the validation set to be used to blindly test the methods, and externally confirm the exactitude of the model. We have additionally included a data processing pipeline to summarize our methodology.

## Results

In dataset 1 (Iran), all the available patient records were used to train the models. The median age of patients was 69 years with an interquartile range (IQR) of 54–78. The minimum and maximum ages were 20 and 98 years, respectively. One hundred fifty-three patients (65.1%) were men, and 82 (34.9%) were women. One hundred five (39.9%) were discharged from the ICU after recovery and 158 (60.1%) patients died. The most frequent comorbidities among the patients were hypertension, diabetes, and cardiovascular disorders in 94, 92, and 86 patients, respectively. Among the 158 deceased patients, neurological disorders were the most prevalent comorbidity (42 patients, 84%). The statistical analysis and the availability of each parameter in our dataset are summarized in Table 2.

**Table 2:** Characteristics of intensive care unit patients with COVID-19 in our data.

	**Survived (*****N*** **=** **105)**		**Died (*****N*** **=** **158)**		**Sig. key:** <0.1 (*), <0.01 (**), <0.001 (***)			
	**Number (%)**	**Available data (%)**	**Number (%)**	**Available data (%)**	**Total (number)**	**X^2^ statistics**	**X^2^ *p*-value**	**Sig**.
Gender		105 (100)		158 (100)		1.70	0.19	
Male	63 (36.4)	..	110 (64.6)	..	173	..	..	
Female	42 (46.7)	..	48 (53.3)	..	90	..	..	
Comorbidity	..	105 (100)	..	158 (100)	..	..	..	
Autoimmune disorder	2 (33.3)	..	4 (66.7)	..	6	0.10	0.74	
Cancer	6 (42.9)	..	8 (57.1)	..	14	0.05	0.82	
Cardiovascular disorder	25 (29.1)	..	61 (70.9)	..	86	4.22	0.04	*
Diabetes mellitus	35 (38.0)	..	57 (62.0)	..	92	0.13	0.71	
Thrombosis	2 (40.0)	..	3 (60.0)	..	5	0.0003	0.99	
Hypertension	32 (34.0)	..	62 (66.0)	..	94	1.35	0.24	
Hepatic failure	2 (40.0)	..	3 (60.0)	..	5	0.0007	0.99	
Neurological disorder	8 (16.0)	..	42 (84.0)	..	50	11.93	<0.001	***
Respiratory disorder	7 (24.1)	..	22 (75.9)	..	29	3.01	0.08	*
	**Median (IQR)**	**Available data (%)**	**Median (IQR)**	**Available data (%)**	**Normal range**	**KS Statistics**	**KS** ***p*****-value**	
Age (years)	58.0 (47.0–73.0)	105 (100)	72.5 (64.0–80.75)	158 (100)	..	0.35	<0.001	***
pH	7.42 (7.375–7.457)	87 (82)	7.4 (7.33–7.441)	129 (81)	7.31–7.41	0.18	0.05	*
pCO_2_ (mm Hg)	38.4 (34.8–45.1)	87 (82)	40.2 (33.9–47.1)	125 (79)	35–40	0.09	0.66	
pO_2_ (mm Hg)	37.05 (25.1–57.425)	86 (81)	39.9 (26.975–56.65)	124 (78)	42–51	0.08	0.81	
HCO_3_ (meq·L)	25.5 (22.825–28.575)	86 (81)	24.2 (21.2–27.55)	123 (77)	22–26	0.15	0.14	
O_2_ saturation (%)	72.7 (48.3–89.2)	85 (80)	73.5 (50.2–88.95)	123 (77)	−2.0 to 2.0	0.08	0.87	
Base excess (mEq/L)	2.2 (−0.55 to 4.65)	87 (82)	0.6 (−3.1 to 3.275)	126 (79)	..	0.18	0.06	*
Total buffer base (mEq/L)	49.1 (46.65–51.75)	87 (82)	47.5 (43.75–50.375)	126 (79)	..	0.20	0.01	*
Base excess in the extracellular fluid (mEq/L)	2.2 (−0.4 to 4.9)	87 (82)	0.35 (−3.175 to 3.75)	126 (79)	..	0.21	0.01	*
White blood cells count (x1000·mm^3^)	7.4 (5.0–11.225)	104 (99)	9.7 (7.1–13.45)	155 (98)	4.0–10.0	0.23	0.002	**
Band (%)	3.0 (2.0–5.5)	23 (21)	3.0 (2.0–6.0)	38 (24)	..	0.05	1	
Segment (%)	78.0 (70.65–83.0)	87 (82)	82.8 (77.05–86.95)	119 (75)	..	0·25	0.002	**
Lymphocyte (%)	14.0 (10.0–20.225)	86 (81)	10.7 (6.85–15.4)	119 (75)	..	0.25	0.002	**
Monocyte (%)	6.0 (4.0–8.5)	45 (42)	5.0 (3.35–7.0)	59 (37)	..	0.17	0.34	
Basophil (%)	0.3 (0.2–0.8)	13 (12)	0.1 (0.0–0.1)	13 (8)	..	0.61	0.01	
Red blood cells count (mill·mm^3^)	4.335 (3.83–4.908)	102 (97)	4.185 (3.64–4.748)	154 (97)	4.2–5.4	0.12	0.28	
Hemoglobin (g·dl)	12.6 (10.95–13.8)	103 (98)	12.2 (10.2–13.75)	155 (98)	12.0–16.0	0.07	0.81	
Hematocrite (%)	37.0 (32.85–41.2)	103 (98)	36.6 (31.45–40.75)	155 (98)	36–46	0.06	0.93	
Mean corpuscular volume (fL)	85.0 (81.4–88.65)	103 (98)	88.0 (84.65–91.9)	155 (98)	77–97	0.24	<0.001	***
Mean corpuscular hemoglobin (Pgm)	28.7 (26.6–29.85)	103 (98)	29.6 (27.8–30.55)	155 (98)	26–32	0.21	0.006	**
Mean corpuscular hemoglobin concentration (%)	33.1 (32.45–34.4)	103 (98)	33.3 (31.95–34.15)	155 (98)	32–36	0.09	0.59	
Platelet count (x1000·mm^3^)	196.0 (151.5–260.0)	103 (98)	179.0 (125.0–255.0)	155 (98)	140–440	0.17	0.04	*
Red cell distribution width (%)	13.95 (13.2–14.825)	88 (83)	14.6 (13.75–16.0)	131 (82)	11.0–16.0	0.23	0.006	**
Platelet distribution width (FL)	12.8 (11.5–14.0)	85 (80)	13.2 (11.4–14.7)	120 (75)	10.0–17.0	0.13	0.32	
Mean platelet volume (FL)	9.7 (9.175–10.5)	84 (80)	10.0 (9.3–10.7)	120 (75)	8.5–12.5	0.13	0.30	
Platelet larger cell ratio (%)	24.4 (19.85–29.3)	83 (79)	26.7 (21.05–30.825)	120 (75)	17–45	0.17	0.07	*
C-reactive protein (mg·l)	48.0 (24.0–48.0)	56 (53)	48.0 (48.0–48.0)	67 (42)	<6	0.23	0.05	*
Erythrocyte sedimentation rate (mm · hr)	42.0 (27.5–68.5)	55 (52)	59.0 (33.75–75.25)	56 (35)	<20	0.24	0.06	*
Albumin level (g·dl)	3.3 (3.0–3.7)	48 (45)	2.9 (2.6–3.2)	71 (44)	3.5–5.5	0.37	<0.001	***
Serum calcium level (mg·dl)	8.8 (8.3–9.2)	72 (68)	8.6 (7.9–9.2)	97 (61)	8.6–10.6	0.13	0.37	
Inorganic P level (mg·dl)	3.3 (2.45–4.4)	59 (56)	4.0 (2.95–5.4)	87 (55)	2.5–5.0	0.20	0.08	
Serum Na level (mg·dl)	137.5 (135.0–140.0)	102 (97)	139.0 (135.0–142.0)	155 (98)	136–145	0.17	0.03	*
Serum K level (mg·dl)	4.3 (3.925–4.6)	102 (97)	4.4 (4.0–4.85)	155 (98)	3.7–5.5	0.11	0.37	
Serum Mg level (mg·dl)	2.25 (2.0–2.5)	66 (62)	2.4 (2.0–2.7)	96 (60)	1.8–2.6	0.14	0.32	
Uric acid level (mg·dl)	6.7 (4.05–9.0)	15 (14)	8.2 (5.95–9.95)	31 (19)	3.4–7.0	0.37	0.10	
Fasting plasma glucose (mg·dl)	124.0 (105.0–177.0)	65 (61)	154.0 (120.5–246.5)	99 (62)	..	0.21	0.04	*
Blood urea nitrogen (mg·dl)	16.0 (11.25–22.5)	102 (97)	30.0 (21.0–52.5)	156 (98)	5.0–23.0	0.47	<0.001	***
Creatinine (mg·dl)	1.1 (0.9–1.4)	102 (97)	1.5 (1.2–2.2)	156 (98)	0.5–1.5	0.31	<0.001	***
Aspartate aminotransferase (IU·L)	40.0 (29.0–55.0)	83 (79)	45.0 (31.5–82.5)	112 (70)	5.0–40.0	0.17	0.10	
Alanine aminotransferase (IU·L)	26.0 (16.0–38.5)	83 (79)	25.0 (18.0–45.0)	113 (71)	5.0–40.0	0.11	0.54	
Lactate dehydrogenase (U·L)	710.0 (561.0–1019.0)	57 (54)	859.0 (623.5–1256.0)	95 (60)	225–500	0.17	0.20	
Creatine phosphokinase (IU·L)	233.0 (89.0–546.5)	59 (56)	204.0 (83.0–434.0)	91 (57)	24–195	0.08	0.90	
Creatine phosphokinase-MB (U·L)	30.0 (22.5–41.0)	35 (33)	30.0 (24.0–49.0)	41 (25)	5–25	0.10	0.96	
Alkaline phosphatase (IU·L)	180.0 (132.5–248.5)	75 (71)	193.0 (155.75–264.25)	96 (60)	64–306	0.12	0.46	
Total bilirubin (mg·dl)	0.7 (0.5–0.9)	46 (43)	0.8 (0.6–1.45)	71 (44)	0.2–1.2	0.25	0.04	*
Direct bilirubin (mg·dl)	0.25 (0.2–0.3)	46 (43)	0.3 (0.2–0.6)	71 (44)	0–0.4	0.27	0.02	*
Prothrombin time (Sec)	14.9 (14.0–16.5)	81 (77)	16.0 (14.3–18.0)	121 (76)	12.0–13.0	0.23	0.009	**
Prothrombin time activity (%)	81.0 (72.0–89.0)	40 (38)	73.0 (54.5–85.5)	51 (32)	85–100	0.29	0.03	*
International normalized ratio (index)	1.2 (1.08–1.3)	81 (77)	1.3 (1.108–1.6)	120 (75)	1.0–1.1	0.29	<0.001	***
Partial thromboplastin time (Sec)	34.0 (30.0–40.0)	81 (77)	36.0 (31.0–45.75)	122 (77)	25–45	0.16	0.12	
D-Dimer (ng·ml)	1513.0 (1071.0–2207.0)	9 (8)	1875.0 (1558.0–5269.0)	11 (6)	..	0.28	0.68	
Interleukin-6 (pg·ml)	115.0 (76.0–147.25)	10 (9)	107.0 (47.0–301.0)	17 (10)	..	0.37	0.27	
Fibrin degeneration product (mg·L)	12.0 (12.0–12.0)	1 (0)	18.0 (18.0–18.0)	1 (0)	..	1	1	
Troponin (ng·L)	227.5 (78.375–1400.5)	12 (11)	49.0 (28.2–1027.0)	21 (13)	..	0.27	0.53	
Fibrinogen (mg·dl)	546.0 (477.5–727.0)	7 (6)	482.5 (226.75–615.75)	18 (11)	308–613	0.38	0.33	
Hemoglobin A1c (%)	7.2 (6.9–7.5)	5 (4)	6.4 (5.5–7.0)	9 (5)	..	0.55	0.22	

SIG. Key: * = <0.1, ** = <0.01, *** = <0.001.

In the RF model, the optimum point between overfitting and efficiency was found by selecting 10 laboratory biomarkers out of 55 with the lowest KS *p*-values and three out of nine comorbidities with the lowest χ^2^ *p*-values, besides demographic characteristics.

The selected numerical parameters for modeling were as follows: age, blood urea nitrogen (BUN), serum creatinine level (Cr), international normalized ratio (INR), serum albumin, mean corpuscular volume (MCV), red cell distribution width (RDW), mean corpuscular hemoglobin (MCH), white blood cell count (WBC), segmented neutrophil count, and lymphocyte count. In addition, selected categorical parameters were gender and a history of neurological, respiratory, and cardiovascular diseases. The distributions of selected numerical (age and biomarkers) and categorical (gender and preexisting conditions) variables are shown in Supplementary Figures 6, 7, respectively.

Based on the ROC curves of the models (Supplementary Figure 3), the RF model outperformed other models and had superior efficiency. The higher efficiency of the RF model is also statistically significant in comparison to the other methods (Supplementary Figure 4). The better performance of RF could be explained by the complexity of the effects of COVID-19 and the varied etiologies underlying the deterioration of COVID-19 patients, for which the non-linear characteristics of the RF model was a more suitable option for predictions than the linear LR model. The RF model could predict a patient's outcome with a sensitivity of 70% and a specificity of 75%, whereas the sensitivity for the LR model was 65% and the specificity was 70%. Evaluation metrics for the models were also confirmed by the metrics reported as the results of the validating models.

By using the LIME technique, variables that provide the most information on the probability of each patient's death were identified. Among the six submodules identified with the highest disparity among 100 patients, albumin, BUN, and RDW were present in five of them. Age, MCH, and creatinine were present in four of the abovementioned submodules. This points out the importance of these measurements in the recorded parameters. Additionally, BUN (in three of these submodules), RDW (in two submodules), and age (in one submodule) were the most decisive ones.

**Figure 3:**
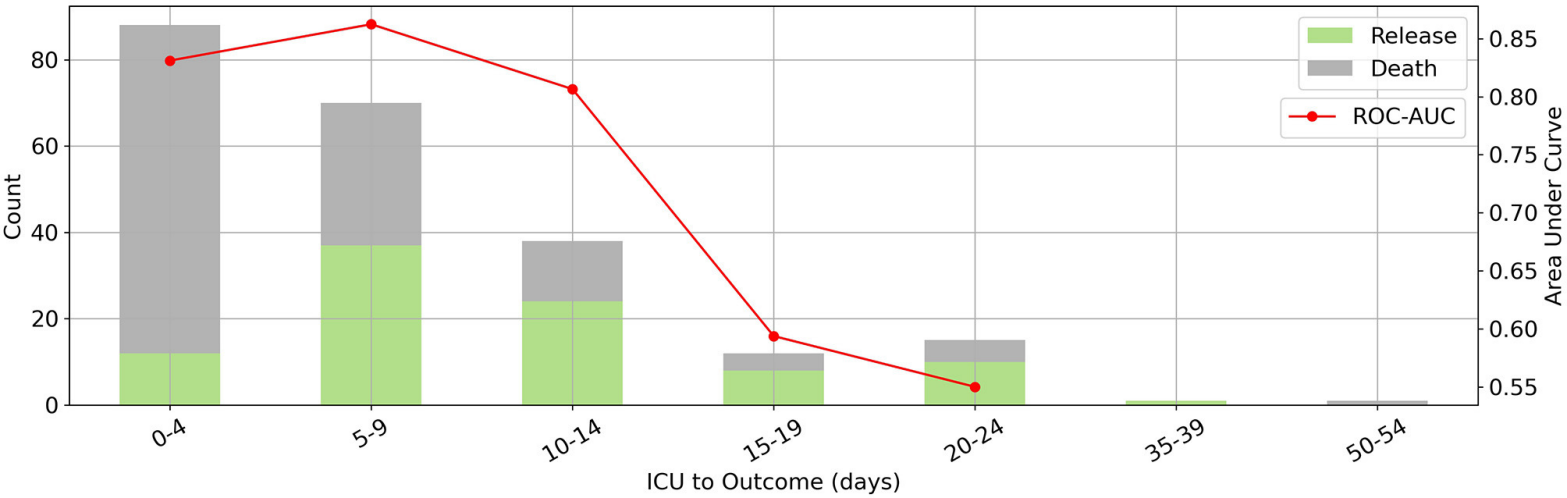
The relation between prediction horizon and performance. Where x-axis denotes days from ICU admission to outcome. Distribution of days between intensive care unit admission and outcome (bars on the left vertical axis) and corresponding random forest model's area under the receiver operating characteristic curve scores for each bin (red line on the right vertical axis). Our model has the best performance to predict outcomes in a 15-day period.

This model could predict a patient's outcome reliably (AUC between 80 and 85) over a 15-day period, as shown in Figure 3. The mortality rate was highest between zero and 4 days. Given that the model was designed for first-day ICU admissions, moving away from this day reduced the accuracy of the predictions and the efficacy of the LIME method for clinical interventions, as expected.

**Figure 4:**
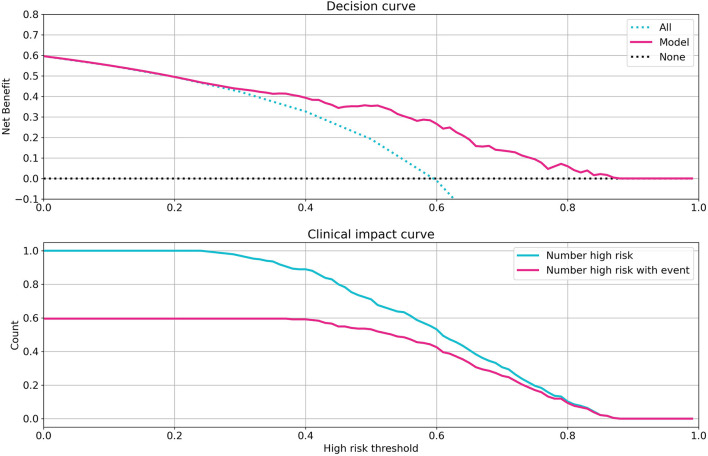
Investigation of clinical impacts and benefits of the model. Decision curve **(Top)** and clinical impact curve **(Bottom)** of the random forest model. The decision curve compares the net benefits of an intervention in three scenarios: intervention for all patients (blue dotted line), intervention for no patients (gray dotted line), and intervention for high-risk patients based on the model prediction (red line). The clinical impact curve compares the number of patients classified as high risk by model and the number of patients with a really poor bad outcome who were classified as high risk, for all possible high-risk thresholds in model prediction from 0 to 1.

To evaluate the clinical capability of the model, the decision curve (DC) and the clinical impact curve (CIC) were investigated (23). The DC framework measures the clinical “net benefit” for the prediction model relative to the current treatment strategy for all or no patients. The net benefit is measured over a spectrum of threshold probabilities, defined as the minimum disease risk at which further intervention is required. Based on the DC, CIC, and on the assumption of the same interventions for high-risk patients, our model indicated a superior or equal net benefit within a wide range of risk thresholds and patient outcomes, as shown in Figure 4.

### Validation of the Model

In order to validate the performance of the model, similar records for 161 patients admitted to ICUs and Emergency Assessment Units were studied to externally confirm the prediction model (from dataset 2, U.K. cohort; see graphical abstract). The same Data preprocessing routine was applied to the additional validation data and ML methods with the same parameters as mentioned in Table 1 were implemented. Models were blindly tested with the external validation data. Evaluation metrics for models are reported in Supplementary Table 3. Reported evaluation metrics indicate a 70% sensitivity for the RF model which accredits the certitude of the model. Validation results ensure the generalizability of the model and guarantee it's applicability for external data containing similar, globally accessible features.

## Discussion

The aim of this study was to develop an interpretable ML model to predict the mortality rate of COVID-19 patients at the time of admission to the ICU. To the best of our knowledge, this is the first study to develop a predictive model of mortality in patients with severe COVID-19 infection at such an early stage using routine laboratory results and demographic characteristics.

Statistical analysis and feature selection tasks were performed merely by considering patients in dataset 1 (Iran dataset), which includes routine laboratory results, past medical histories and demographic characteristics, leading to selection among accessible and measurable predictors.

The most decisive parameters based on the two-sample KS test were, in decreasing order of importance, increased BUN, Cr, INR, MCV, WBC, segmented neutrophils count, RDW, MCH, and decreased albumin and lymphocyte levels. Moreover, based on a χ^2^-test, age, gender, and a history of neurological, cardiovascular, and respiratory disorders were identified as parameters with high predictive values. Multicollinearity might affect the performance of the models and result in redundancy. Hence, variance inflation factor was calculated to find and remove highly correlated predictors. Selected predictors along with their references in the literature are listed in Table 3.

**Table 3:** Predictors with the highest predictive value, selected in this study, along with studies referring to them.

**Predictor**	**Description**	**Literature references**
Gender	Sex-dependent differences in clinical manifestation	(24, 25)
Age	Higher age affects COVID-19 poor outcomes	(25, 26)
Blood Urea Nitrogen	Assumed highest weights for prognosis	(27, 28)
Creatinine	A lower creatinine clearance levels increases the mortality	(29)
INR	INR >1.3 significantly increases mortality	(30)
Albumin	Assumed highest weights for prognosis	(27)
WBC	Abnormal white blood cell count increases mortality	(31)
Neutrophil count	affects COVID-19 poor outcomes	(32)
Lymphocyte count	Lymphocytes <10% increases mortality	(30)
RDW	RDW >14.5% increases mortality	(30)
MCH	Abnormal MCH increases mortality	(33)
Neurological disorders	Affects the COVID-19 outcome	(34)
Cardiovascular disorders	Affects the COVID-19 outcome	(34)
Respiratory disorders	Affects the COVID-19 outcome	(34)

A number of studies have investigated the risk factors affecting COVID-19 infections (34, 35). Elevated inflammatory cytokines such as interleukin-6 (IL-6), granulocyte colony-stimulating factor (G-CSF), interferon gamma-induced protein 10 (IP-10), and interferon (IFN)-γ have been proposed as poor prognostic factors for COVID-19 patients (36–39). These markers, however, are not usually used as predictors of the severity of disease in clinical practice. Although using these cytokines in modeling may enable a more accurate prediction of the severity of COVID-19 infection, doing so impedes the model's clinical application, as most of the cytokines are not routinely checked at presentation to the ICU. In contrast, all 10 laboratory biomarkers identified in our model are commonly measured and are available to most clinical laboratories. Thus, the DC and CIC analyses indicated the notable clinical benefit of our model especially in a situation characterized by resource scarcity.

Only patients who had at least seven of the 10 selected biomarkers have been included in the training phase of the modeling and missing parameters were imputed using k-NN based on the data. As can be seen in the models' ROC curve, the RF algorithm outperformed other methods in predicting the outcome. Significance of this difference has been investigated using DeLong's test. Superior proficiency of the RF model is mainly due to the non-linear correlation between variables, manifesting the complexity of the problem.

Since a part of the data itself has been used for feature selection and a 10-fold cross-validation algorithm has been implemented to the data, an additional external validation was conducted to confirm the model's performance. Models were blindly tested with validation data, including records of measurements of the selected predictors for 161 patients, taken out of dataset 2 (U.K.).

Results of the validation assure that the model we developed could be applied globally and predict mortality of the patients with severe COVID-19 infection solely with universally accessible parameters (Table 2). As a result, physicians and healthcare systems are able to utilize this model, confident about high sensitivity and specificity in the outcome.

The application of the LIME-SM method allowed us to determine a patient-specific marker set that each patient's prognosis is based on. This technique explains the predictions by perturbing the input of data samples and evaluating the effects. The output of LIME is a list of features, reflecting each feature's contribution to a given prediction. Understanding the “reasoning” of the ML model is crucial for increasing physicians' confidence in selecting treatments based on the prognosis scores. Using the LIME method, the significance of variables with high predictive value was determined for each prediction made for an individual. The evaluation of the variables in the individual's personalized prediction can lead to supportive measures and help determine treatment strategies according to the interpretation of the individual prognosis.

As severe COVID-19 may result from various underlying etiologies, our model can help categorize patients into groups with distinct clinical prognosis, thus allowing personalized treatments. In addition to targeted therapies, the differentiation between patients may reveal disease mechanisms that coincide or that occur under specific preexisting conditions. Future cohort studies could explore these assumptions with increased sample sizes.

In this study, hypoalbuminemia and renal function were identified as the main factors with high predictive values for the model. These findings are in agreement with recent results showing that hypoalbuminemia is an indicator of poor prognosis for COVID-19 patients (40). It is well-documented that endogenous albumin is the primary extracellular molecule responsible for regulating the plasma redox state among plasma antioxidants (40). Moreover, it has been shown that albumin downregulates the expression of the angiotensin-converting enzyme 2 (ACE2) which may explain the association of hypoalbuminemia with severe COVID-19 (41). Intravenous albumin therapy has been shown to improve multiple organ functions (42). Therefore, early treatment with human albumin in severe cases of COVID-19 patients before the drop in albumin levels might have positive outcomes and needs to be further investigated.

Furthermore, increased levels of BUN and Cr are observed in our study, which is an indication of kidney damage. An abrupt loss of kidney function in COVID-19 is strongly associated with increased mortality and morbidity (43). There are multiple mechanisms supporting this association (44, 45).

One of the findings of this study is the identification of RDW (a measure of the variability of the sizes of RBCs) as an influential parameter. This result is in line with recently published reports (46). Elevated RDW, known as anisocytosis, reflects a higher heterogeneity in erythrocyte sizes caused by erythrocyte maturation and degradation abnormalities. Several studies have found that elevated RDW is associated with inflammatory markers in the blood such as IL-6, tumor necrosis factor-α, and CRP, which is common in severely ill Covid-19 patients (44). These inflammatory markers could disrupt the erythropoiesis by directly suppressing erythroid precursors, promoting apoptosis of precursor cells, and reducing the bioavailability of iron for hemoglobin synthesis.

Yan et al. recently identified LDH, lymphocyte, and high-sensitivity C-reactive protein (hs-CRP) as predictors of mortality in COVID-19 patients during their hospitalization. The blood results of hospitalized patients on different days after the initial ICU admission were used for their model (45). Since our goal was the prediction of mortality risk as early as possible for ICU patients, this limited us to using only the laboratory results on day 0, in contrast. For patients with severe COVID-19 infection, early decision-making is critical for successful clinical management. Additionally, laboratory results from other days may not always become available. We also identified lymphocyte count as a predictor of mortality, as in the previous study; however, CRP levels and LDH did not reach statistical significance.

Although IL-6 has been found to be a good predictor of disease severity by other studies, it did not reach statistical significance in our model (47). IL-6 had a considerable KS statistical value, but because of the high number of missing values, its *p*-value was not significant compared to other markers. The fact that IL-6 is not always measured upon ICU admission is precisely why it is not suitable for our purposes.

In similar studies the impact of laboratory values was assessed. Booth et al. recruited two ML techniques, LR and SVM to design a prediction model for COVID-19 severity among 26 parameters. They indicated CRP, BUN, serum calcium, serum albumin, and lactic acid as the top five highest-weighted laboratory values. Their analysis showed that the SVM model displayed 91% sensitivity and specificity (AUC 0.93) for predicting mortality (27). In another study, Guan et al. used an ML algorithm to predict COVID-19 mortality retrospectively. They showed that CRP, LDH, ferritin, and IL-10 were the most important death predictors with a sensitivity of 85% (48). Zoabi et al. developed a ML-based predicting model that evaluate eight binary features: sex, age, known contact with an infected person, and five initial clinical symptoms including headache, sore throat, cough, fever, and shortness of breath. They showed that their model can predict the COVID-19 infection with 87.30% sensitivity and 71.98% specificity (49).

The missingness indicator of some markers in both LR and RF models has an impact on the predictions based on the regression coefficient and LIME, which can be the result of the model compensating for the imputation error. However, the missingness indicator may also indicate the existence of bias in biomarker reporting (50). Such biases (e.g., sampling bias) are an inevitable part of retrospective studies. They can be addressed using domain-adaptation techniques such as correlation alignment (CORAL) in future studies using additional data (51, 52). Another limitation of this study may be the lack of an objective criterion for ICU admission. Moreover, different treatment strategies can change the survival outcome for patients who may have had similar profiles when admitted to the ICU. In future studies, the accuracy of this model may be further improved by adding chest imaging data and by using a larger dataset. Possible targets for our ML framework include the prediction of other crucial information such as the patients' need for mechanical ventilation, the occurrence of cytokine release syndrome, the severity of acute respiratory disease syndrome, the cause of death, and the right treatment strategy.

In conclusion, we evaluated 66 parameters in COVID-19 patients at the time of ICU admission. Of those parameters, 15 metrics with the highest prediction values were identified: gender, age, BUN, Cr, INR, albumin, MCV, RDW, MCH, WBC, segmented neutrophil count, lymphocyte count, and past medical history of neurological, respiratory, and cardiovascular disorders. In addition, by using the LIME-SP method, we identified different submodules clarifying distinct clinical manifestations of severe COVID-19. The ML model trained in this study could help clinicians determine rapidly which patients are likely to have worse outcomes, and given the limited resources and reliance on supportive care allow physicians to make more informed decisions.

## Data Availability Statement

The original contributions presented in the study are included in the article/Supplementary Material, further inquiries can be directed to the corresponding author/s.

## Ethics Statement

Written informed consent was obtained from the individual(s) for the publication of any potentially identifiable images or data included in this article.

## Author Contributions

EJ, AA, SaJ, NM, and NT: conceptualization, methodology, project administration, writing—original draft, writing—review, and editing. SS: methodology, project administration, writing—review, and editing. AZ, HE, SeJ, AD, AB, MS, and MJ: data curation, investigation, writing—original draft, writing—review, and editing. All authors contributed to the article and approved the submitted version.

## Funding

IORD was supported by the Oxford NIHR Biomedical Research Centre.

## Conflict of Interest

The authors declare that the research was conducted in the absence of any commercial or financial relationships that could be construed as a potential conflict of interest.

## Publisher's Note

All claims expressed in this article are solely those of the authors and do not necessarily represent those of their affiliated organizations, or those of the publisher, the editors and the reviewers. Any product that may be evaluated in this article, or claim that may be made by its manufacturer, is not guaranteed or endorsed by the publisher.

## Abbreviations

ACE2: Angiotensin-Converting Enzyme 2
AI: Artificial Intelligence
BUN: Blood Urea Nitrogen
COVID-19: coronavirus disease of 2019
CIC: clinical impact curve
Cr: creatinine
CRP: C reactive protein
DC: decision curve
ICU: Intensive care unit
INR: International Normalized Ratio
IFN: interferon
IL-6: Interleukin 6
IQR: interquartile range
KS: Kolmogorov- Smirnov
LR: Logistics regression
LIME: local interpretable model-agnostic explanation
LIME-SP: local interpretable model-agnostic explanation submodular-pick
ML: Machine learning
MCH: mean corpuscular hemoglobin
MCV: mean corpuscular volume
RF: Random forest
RDW: Red blood cell distribution width
ROC: receiver operating characteristic curve
RT-PCR: reverse transcription-polymerase chain reaction
WBC: white blood cells count.
