# Supplementary material for: Using Machine Learning to Predict Mortality for COVID-19 Patients on Day 0 in the ICU
Source: Front Digit Health. 2022 Jan 13;3:681608. doi: 10.3389/fdgth.2021.681608 (PMC8792458; doi:10.3389/fdgth.2021.681608)
Supplement: Supplementary file 1 [file Data_Sheet_1.docx]

| **Supplementary Fig. 1. Result of two-sample Kolmogorov-Smirnov test for all numerical parameters.** |
| --- |
| 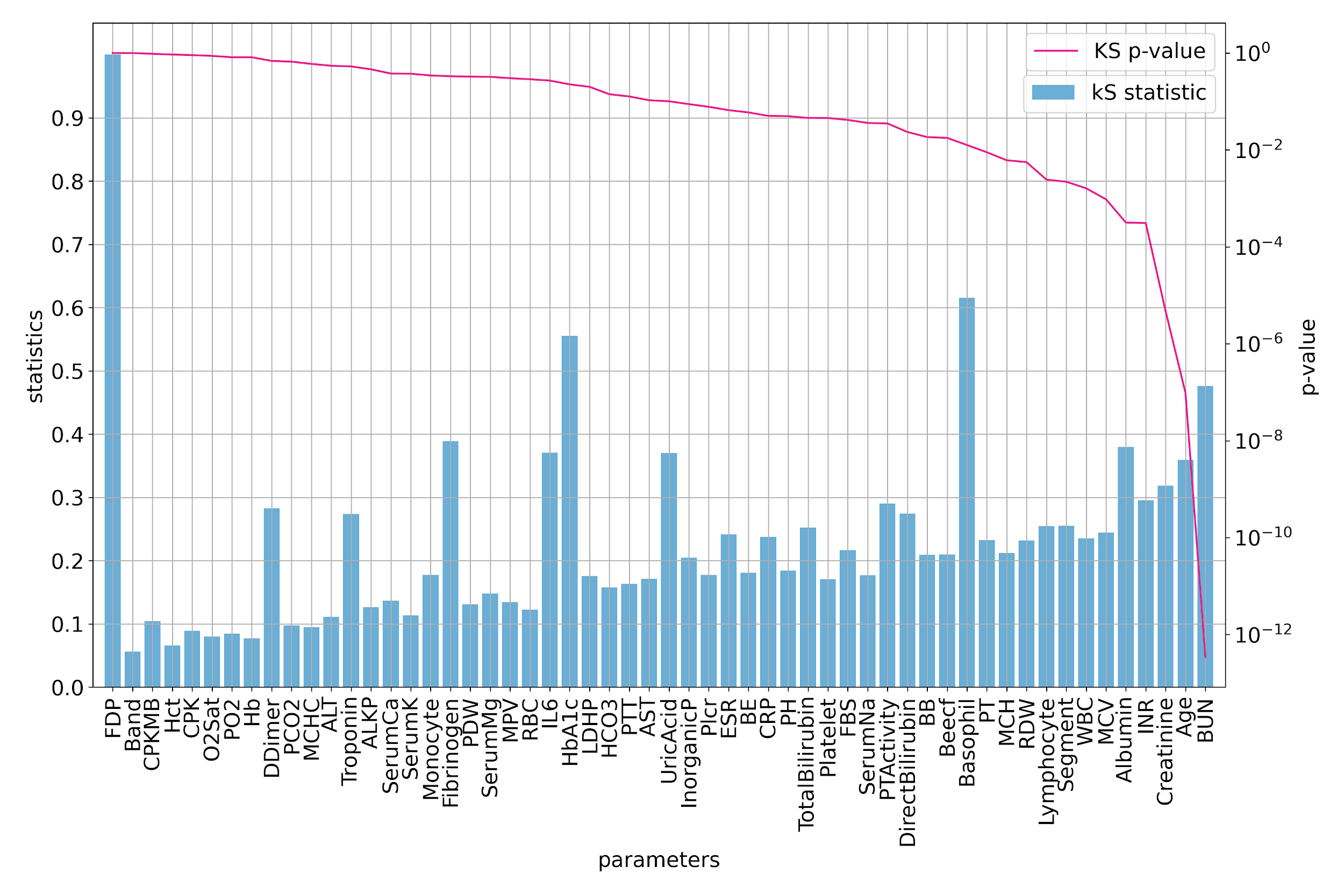 |
| Supplementary Fig. 1. 11 out of 56 numerical features with least Kolmogorov-Smirnov P-values are used for modeling. |

| **Supplementary Fig. 2.** **Result of 𝓧^2^ test for all categorical parameters**. |
| --- |
| 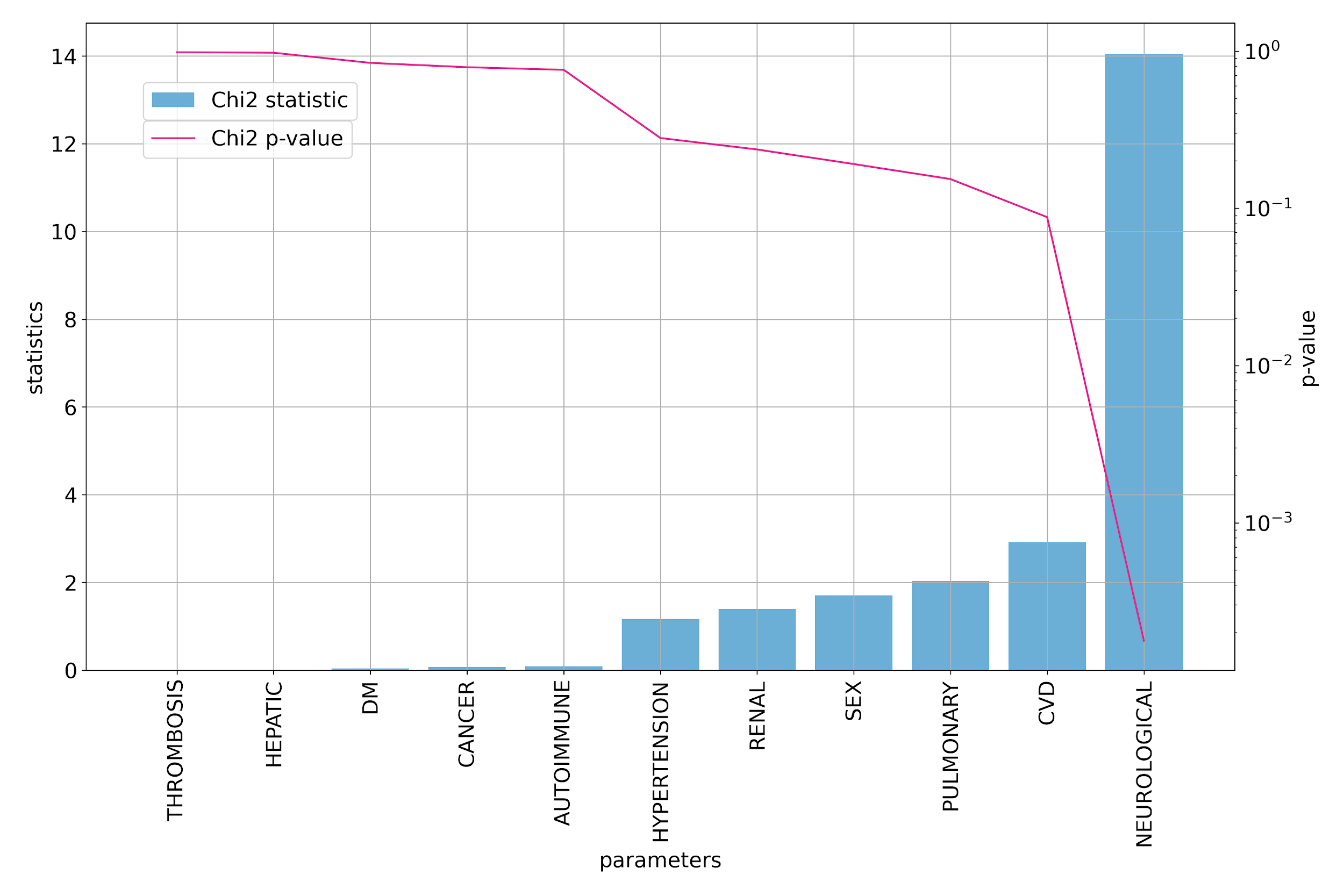 |
| Supplementary Fig. 2. four out of 56 categorical features with least Kolmogorov-Smirnov P-values are used for modeling. |

| **Supplementary Fig. 3. ROC curves and corresponding AUCs for machine learning methods in each iteration.** |
| --- |
| **a)**  **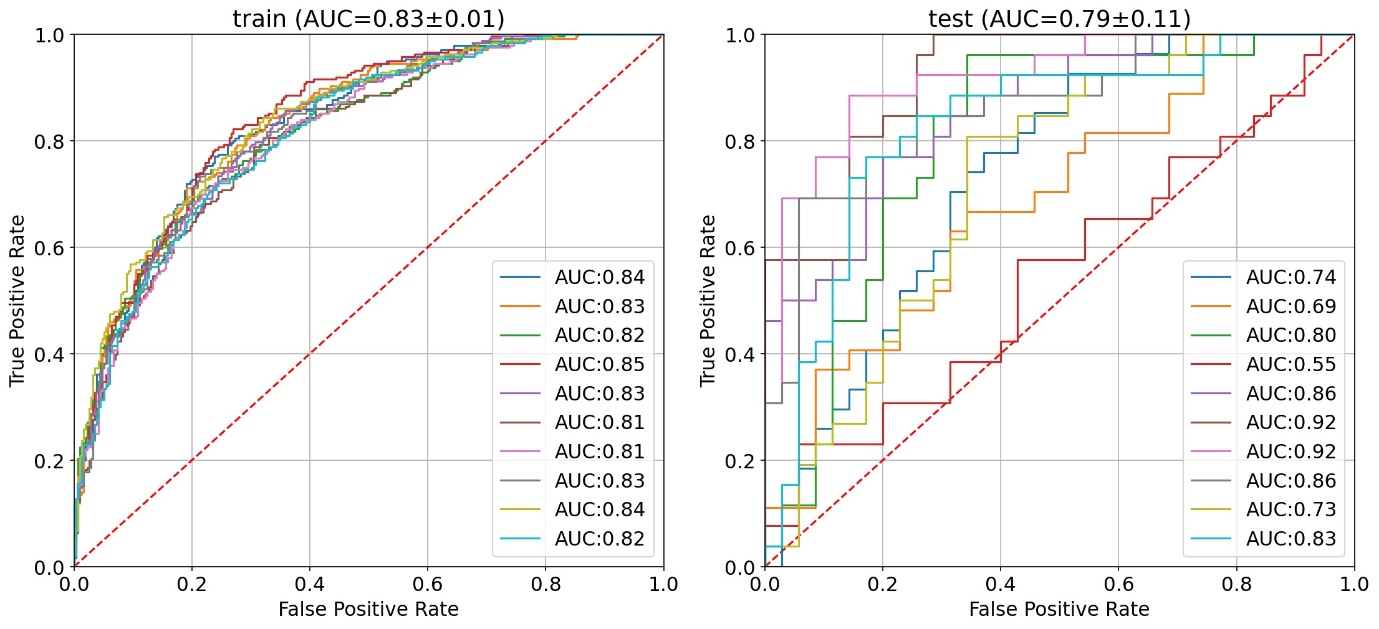**  **b)**  **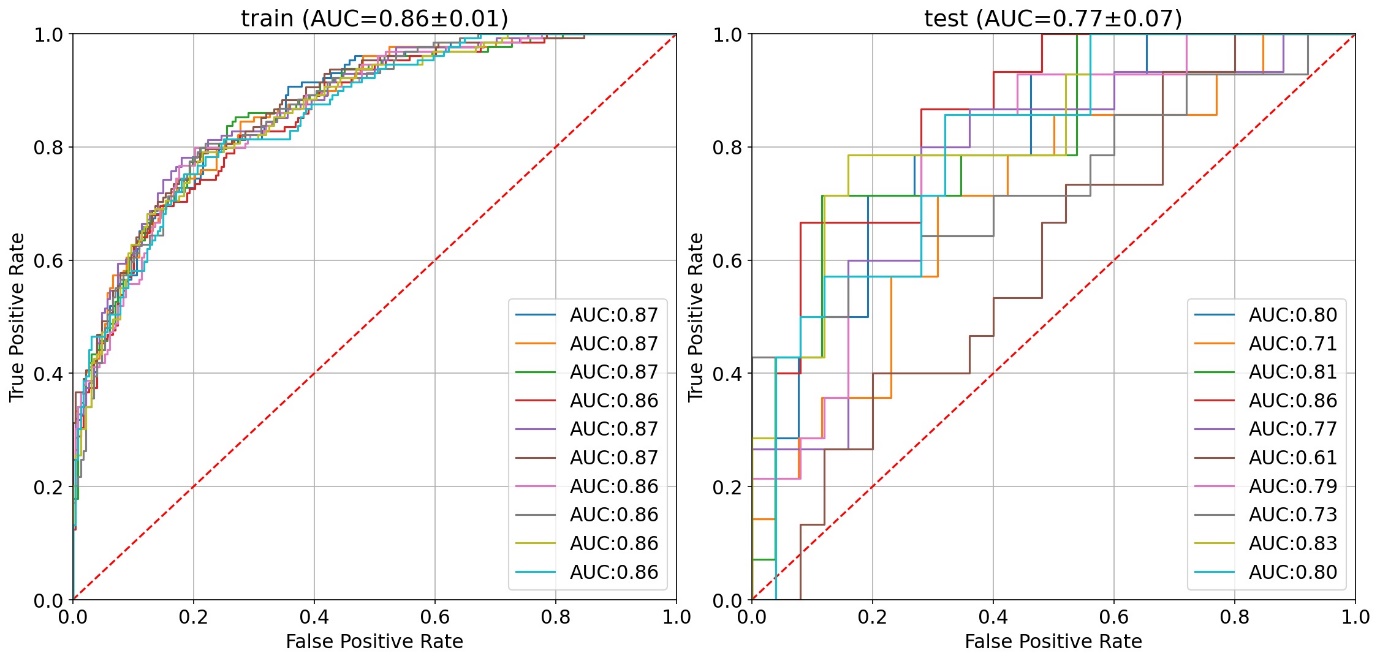c)**  **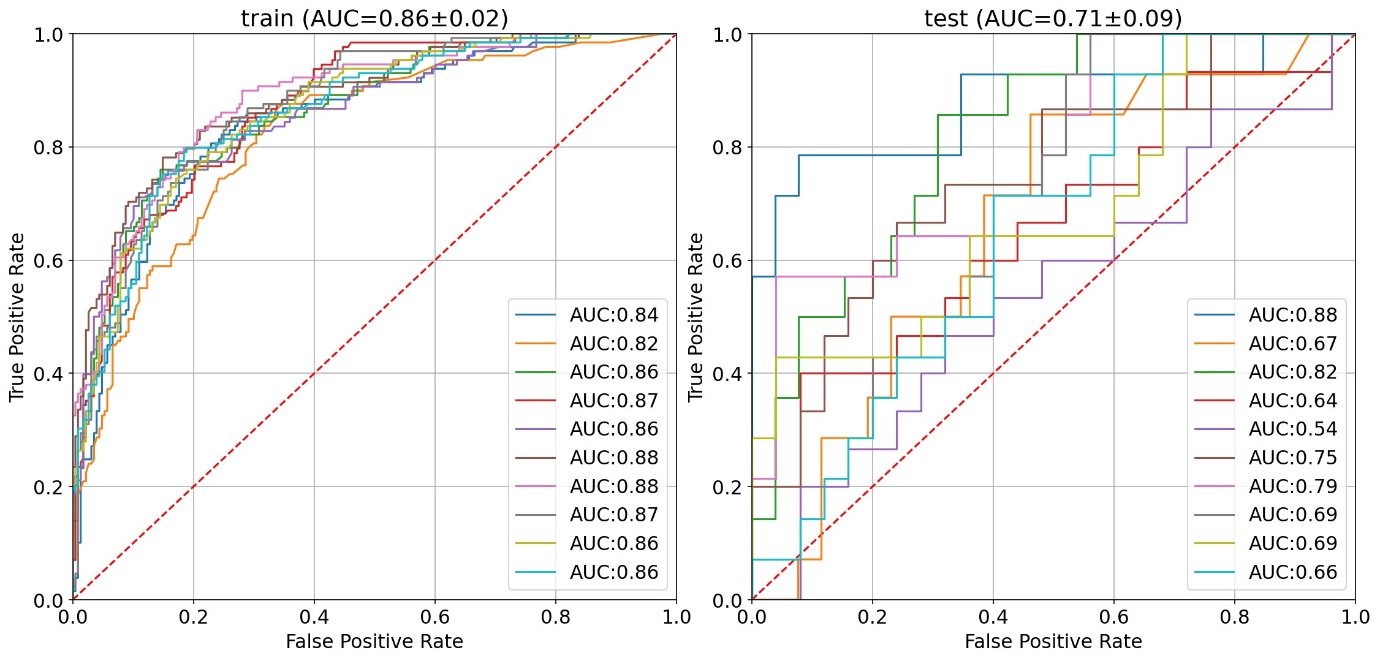d)**  **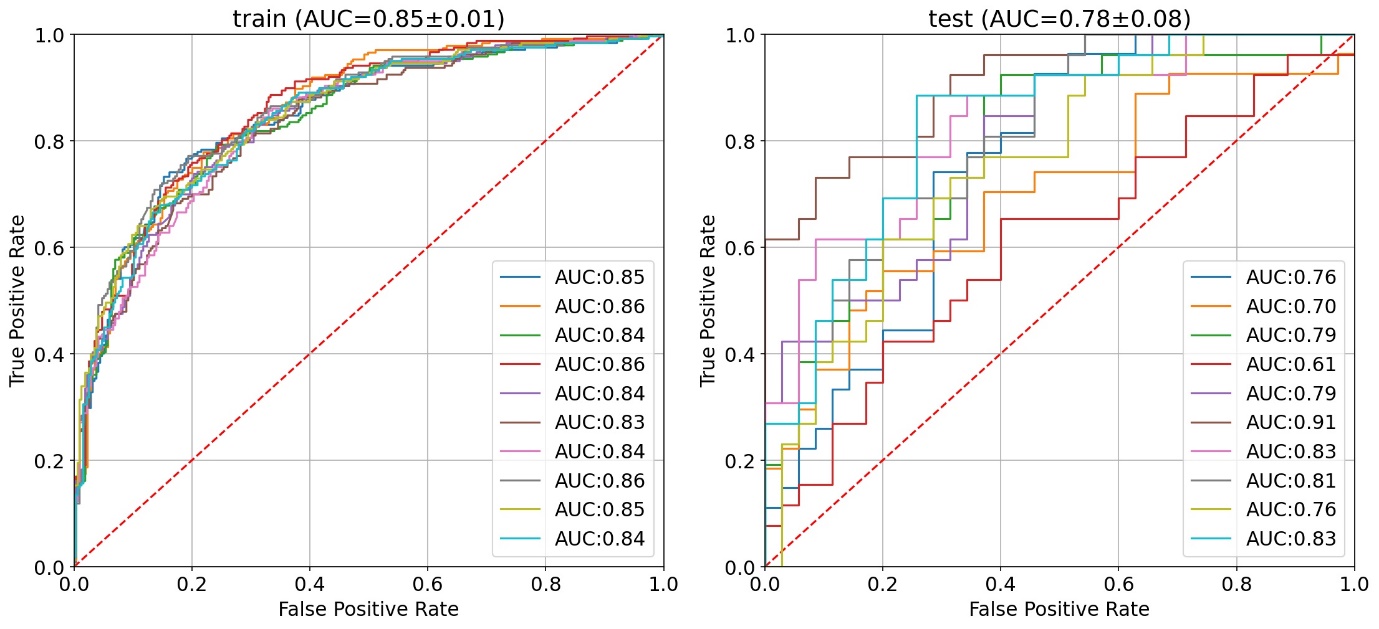**  **e)**  **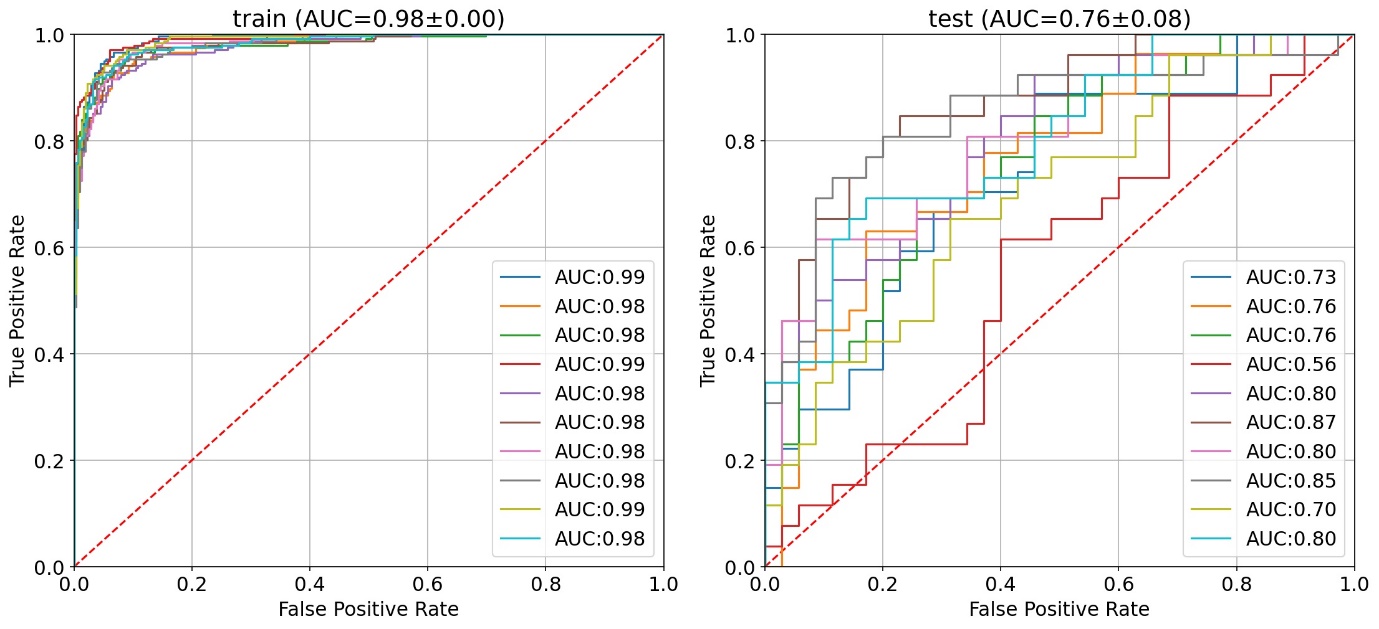** |
| Supplementary Fig. 3. ROC curves for random forest (a), logistic regression (b), gradient boosting classifier (c), support vector machine classifier (d), and artificial neural network (e) methods for training and test sets for each cross-validation iteration and corresponding area under the curve (AUC)s. Error bars represent standard deviation of ROC-AUCs over cross-validation iterations. |

| **Supplementary Fig. 4. DeLong's test and *p*-values** |
| --- |
| **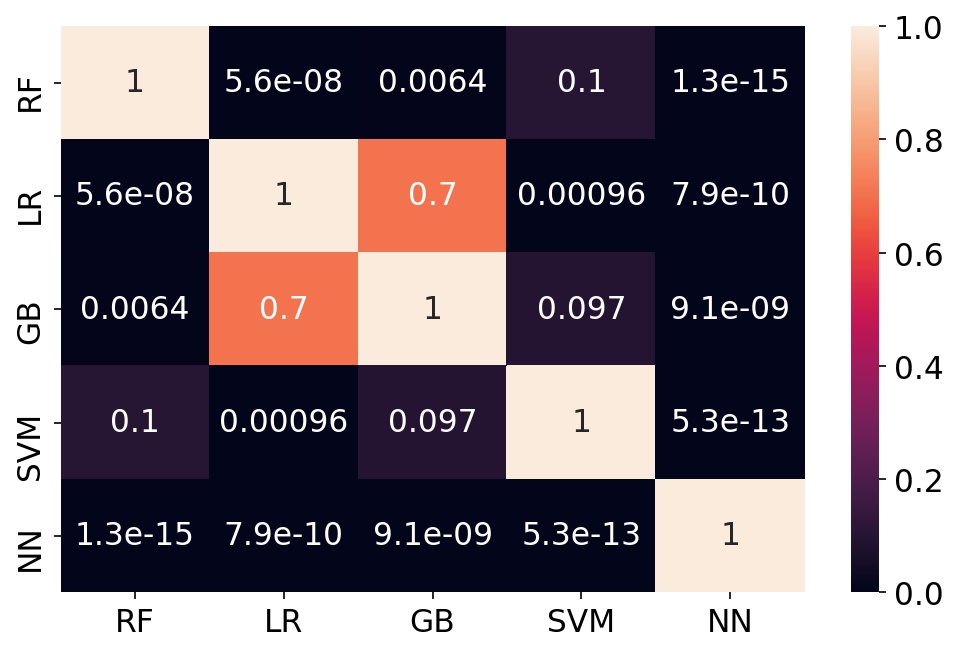** |
| Supplementary Fig. 4. DeLong's test for comparison of the different methods and corresponding *p*-values. |

| **Supplementary Fig. 5. Regression coefficients of the logistic model.** |
| --- |
| 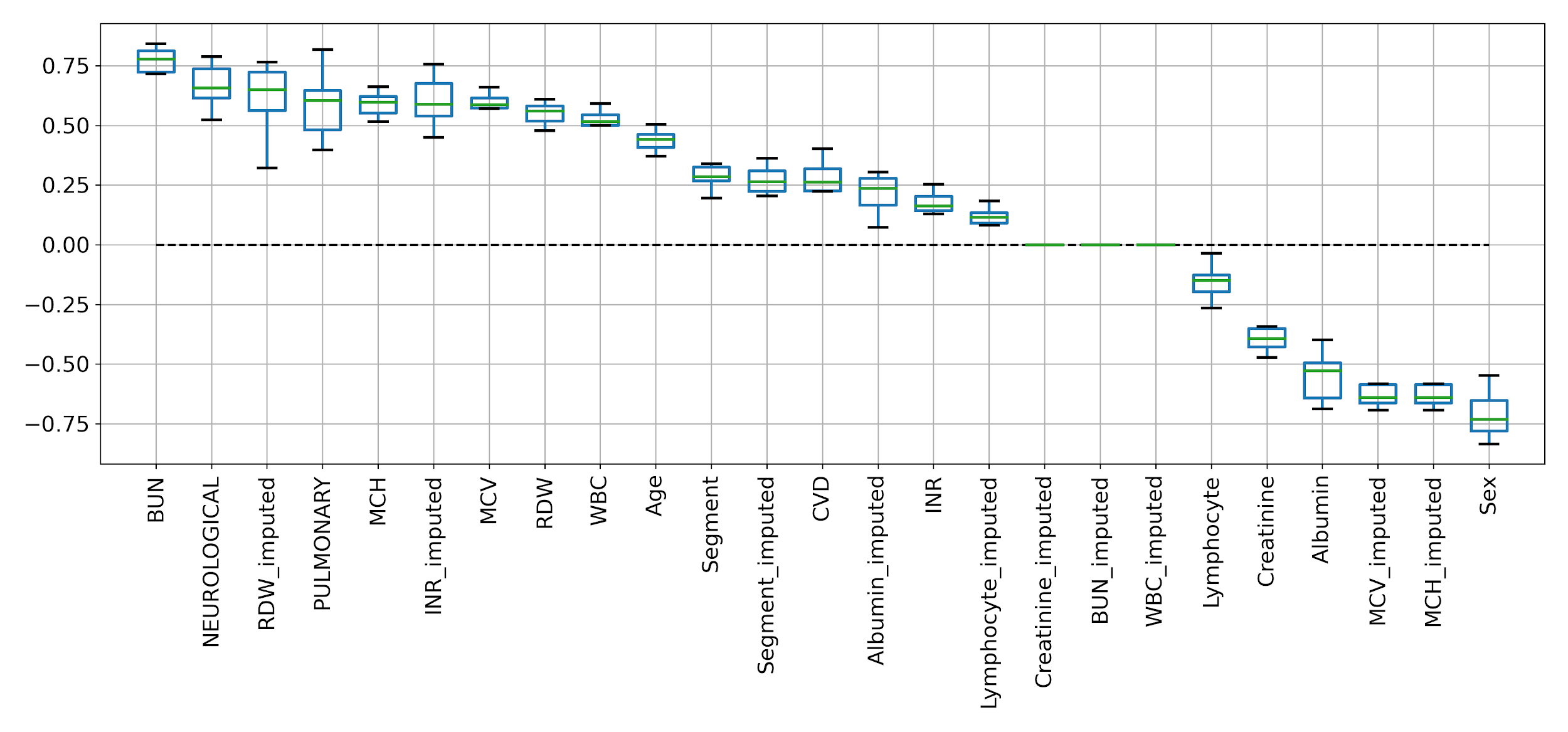 |
| Supplementary Fig. 5. Features with higher coefficient absolute value are more crucial in the logistic model prediction. |

| **Supplementary Fig. 6. Distribution of selected numerical variables.** |
| --- |
| 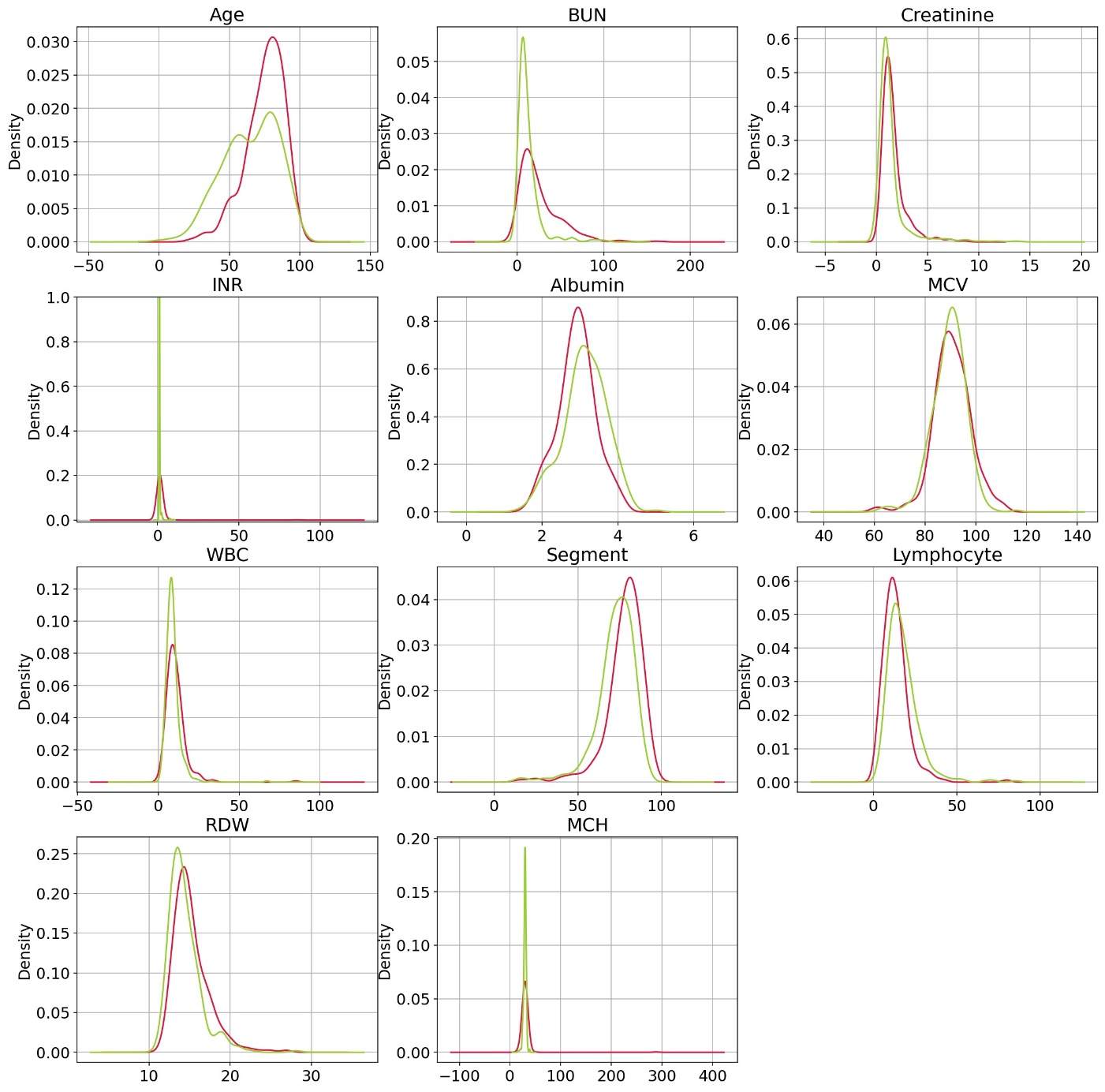 |
| Supplementary Fig. 6. Comparison of categorical variables between released (green) on dead (red) patients. |

| **Supplementary Fig. 7.** **Distribution of selected categorical variable.** |
| --- |
| 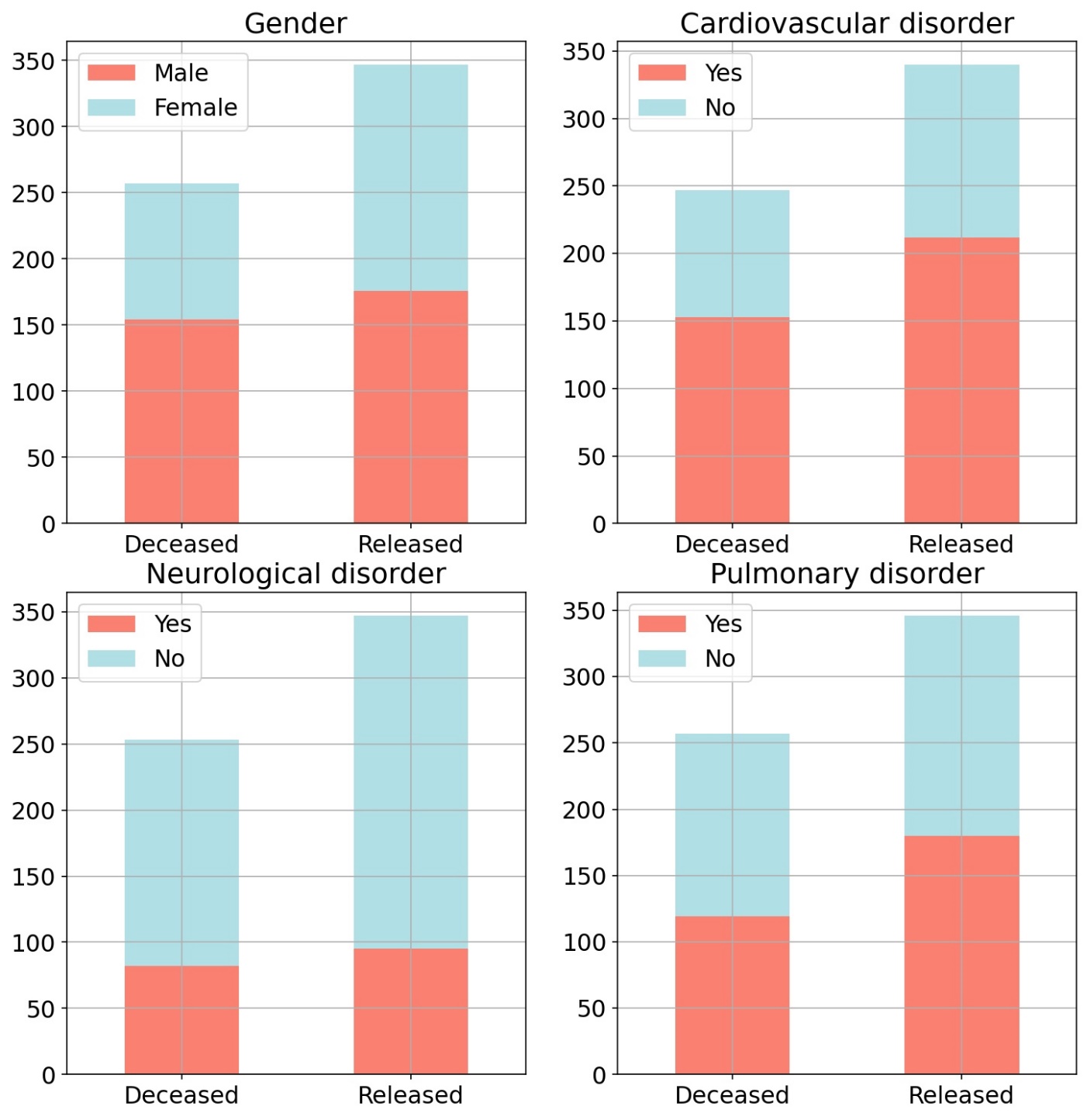 |
| Supplementary Fig. 7. Comparison of categorical variables between released and dead patients. |

**Supplementary Table 1. Variance Inflation Factor (VIF) for predictors. A cut-off of 10 has been used to identify highly correlated features.**

| Predictor | Variance Inflation Factor |
| --- | --- |
| Sex | 1.622 |
| Cardiovascular disorders | 2.578 |
| Neurological disorders | 1.461 |
| Pulmonary disorders | 2.308 |
| Age | 1.315 |
| Blood Urea Nitrogen | 1.935 |
| Creatinine | 1.652 |
| INR | 1.015 |
| Albumin | 1.311 |
| MCV | 1.31 |
| White Blood Cells | 1.143 |
| Segmented Neutrophils | 3.407 |
| Lymphocytes | 3.445 |
| RDW | 1.227 |
| MCH | 1.077 |
| Bilirubin | 1.144 |

**Supplementary Table 2. Evaluation metrics for each machine learning method. Precision and recall metrics were reported as weighted average of metrics over two classes, with respect to the size of the classes.**

| Method | Precision (weighted avg.) | Recall (weighted avg.) | Accuracy |
| --- | --- | --- | --- |
| Random forest | 0.79 | 0.79 | 0.79 |
| Logistic regression | 0.79 | 0.77 | 0.74 |
| Gradient boosting | 0.53 | 0.62 | 0.53 |
| Support vector machine | 0.78 | 0.77 | 0.77 |
| Artificial neural network | 0.69 | 0.69 | 0.69 |

**Supplementary Table 3. Evaluation metric and results of blindly testing the performance of the models over validation dataset.**

| Method | Precision (weighted avg.) | Recall (weighted avg.) | Accuracy | ROC-AUC (±standard deviation) |
| --- | --- | --- | --- | --- |
| Random forest | 0.69 | 0.70 | 0.67 | 0.77 (±0.01) |
| Logistic regression | 0.74 | 0.72 | 0.69 | 0.80 (±0.01) |
| Gradient boosting | 0.79 | 0.68 | 0.59 | 0.75 (±0.03) |
| Support vector machine | 0.73 | 0.72 | 0.70 | 0.78 (±0.01) |
| Artificial neural network | 0.69 | 0.70 | 0.68 | 0.78 (±0.01) |
